# Gene Variations in RNA Modification Pathway Linked to Poor Survival After Gastric Cancer Surgery

**DOI:** 10.3390/genes17080941

**Published:** 2026-08-12

**Authors:** Kağan Gökçe, Mehrdad Sheikhvatan

**Affiliations:** 1 Department of General Surgery, Faculty of Medicine, Surgical Oncology, Istanbul Okan University, Istanbul 34959, Turkey; kgngkc@hotmail.com; 2 Department of Medical Biology and Genetics, Faculty of Medicine, Istanbul Okan University, Istanbul 34959, Turkey; 3 Department of Medical Genetics, Faculty of Medicine, Tehran University of Medical Sciences, Tehran 1416753955, Iran

**Keywords:** m6A modification, gastric cancer, gastrectomy, genetic polymorphism, overall survival, disease-free survival

## Abstract

Background/Objectives: N6-methyladenosine (m6A) RNA modification is a major epitranscriptomic regulator of mRNA stability and translation. Genetic variants in the m6A pathway may influence gastric cancer progression, but their prognostic significance after curative gastrectomy remains uncertain. Methods: This retrospective study included 226 patients with gastric adenocarcinoma who underwent curative gastrectomy. Single nucleotide polymorphisms in five m6A-related genes (METTL3, METTL14, FTO, ALKBH5, and YTHDF1) were genotyped. Overall survival (OS) and disease-free survival (DFS) were analyzed using Kaplan–Meier and log-rank tests. Multivariable Cox regression was performed after adjustment for age, sex, tumor stage, lymph node status, and adjuvant therapy. Results: During a median follow-up of 38 months, 78 patients (34.5%) died and 92 (40.7%) experienced recurrence. High-risk m6A genotypes were associated with significantly poorer survival. The 5-year OS was 68.2% in the low-risk group versus 41.5% in the high-risk group (*p* < 0.001), while 5-year DFS was 61.4% versus 36.8% (*p* < 0.001). High-risk genotypes independently predicted worse OS (HR 1.87, 95% CI 1.28–2.74; *p* = 0.001) and DFS (HR 1.94, 95% CI 1.36–2.78; *p* < 0.001). Patients with the highest genetic risk scores had the poorest outcomes, particularly those with stage III–IV disease. Conclusion: Genetic variants in the m6A RNA modification pathway are independently associated with poorer survival after curative gastrectomy and may serve as prognostic biomarkers for risk stratification and personalized management in gastric cancer.

## 1. Introduction

Even with improvements in surgery and perioperative care, gastric cancer continues to be one of the top causes of cancer-related deaths around the globe. Gastrectomy with curative intent provides the greatest probability for survival, yet there are many instances where there are still recurrences and mortalities among patients who undergo surgery [1,2]. The conventional predictors of prognosis, such as the tumor’s staging, involvement of the lymph nodes, and histopathology, give valuable but insufficient insights into what may happen with patients with gastric cancer [3,4]. There is now an increasing demand to look for new markers to help predict the patient outcomes.

**Figure 1:**
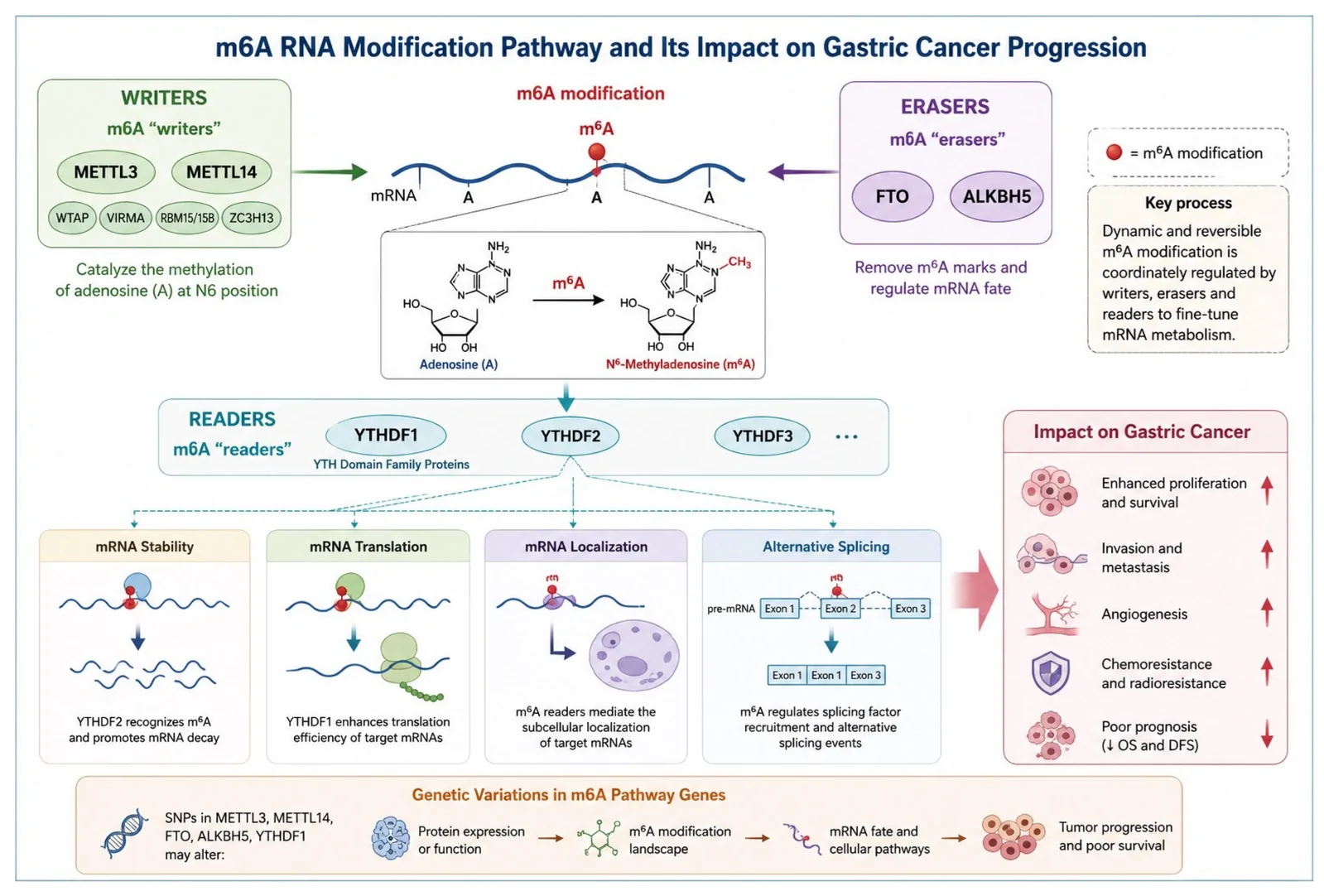
Schematic representation of the m6A RNA modification pathway and its potential role in gastric cancer progression. The diagram summarizes the reported functions of m6A “writers” (METTL3 and METTL14), “erasers” (FTO and ALKBH5), and the “reader” (YTHDF1) based on published experimental studies. The illustration is intended to provide biological context for the investigated polymorphisms and does not represent functional mechanisms directly demonstrated in the present study.

Epitranscriptomics has recently gained prominence as an important factor involved in gene regulation in cancer research. Among several types of RNA modification, N6-methyladenosine (m6A) is the most common type of internal RNA modification in eukaryotes [5,6]. It has a significant role in regulating the processes of splicing, stabilization, localization, and translation of mRNA [7]. The m6A modification process is regulated by the expression of proteins known as “writers,” “erasers,” and “readers.” The abnormal regulation of these protein factors has been linked to the development and progression of cancer, including gastric cancer [8,9]. A schematic overview of the m6A RNA modification pathway and the genes investigated in the present study is presented in Figure 1.

Whereas prior investigations have mainly concentrated on the expression profile of m6A-associated genes, recent findings indicate that genetic variations, particularly SNPs, could potentially regulate the activity of these genes and thereby impact cancer phenotypes [10,11,12]. These genetic changes may lead to modifications in m6A and subsequently modulate the activity of oncogenic or tumor-suppressive signaling pathways [13,14]. Nevertheless, the role of genetic variations in affecting patients’ postoperative survival has yet to be explored thoroughly.

Although numerous studies have demonstrated that aberrant expression of m6A writers, erasers, and readers contributes to gastric cancer initiation, progression, metastasis, and therapeutic resistance [15,16], considerably less attention has been directed toward inherited genetic variation within these regulatory genes. Emerging evidence suggests that single-nucleotide polymorphisms (SNPs) in m6A-related genes, particularly *FTO* and *ALKBH1*, may influence individual susceptibility to gastric cancer by altering m6A-mediated epigenetic regulation and gene expression [17,18]. Nevertheless, comprehensive investigations evaluating the prognostic significance of germline polymorphisms across multiple m6A regulatory genes remain limited. This knowledge gap underscores the need for systematic evaluation of functional variants in m6A pathway genes to clarify their contribution to survival outcomes and to identify novel prognostic biomarkers for precision oncology in gastric cancer.

Considering these facts, the purpose of the current study was to evaluate the correlation between the SNPs selected from key m6A pathway genes and the survival prognosis of patients with gastric cancer following surgical intervention. Another important objective of our study was to calculate the genetic risk score, considering the effects of various genetic variants together, to improve prognostic prediction among such patients.

## 2. Materials and Methods

### 2.1. Study Design and Patient Population

The present study involved a group of patients with histologically proven adenocarcinoma of the stomach who had undergone a curative operation between January 2015 and December 2020 at the referral center. Altogether, 226 patients who had a complete collection of clinicopathologic data, follow-up information, and biological samples for molecular analysis were included in the study. Patients with metastatic disease at the time of diagnosis, history of previous cancer disease, preoperative treatment without surgery, or incomplete medical records were not included in the cohort.

To evaluate the clinical characteristics and survival differences between patients with the most distinct genetic risk profiles, additional analyses were performed using only the lowest and highest tertiles of the genetic risk score. Patients in the intermediate tertile were excluded from these analyses to maximize discrimination between risk groups. Consequently, these comparisons included 150 patients (75 in the low-risk tertile and 75 in the high-risk tertile), whereas analyses evaluating dose–response relationships were performed using all 226 patients categorized into three tertiles.

### 2.2. Ethical Considerations

The study protocol was approved by the institutional ethics committee, and all procedures were conducted in accordance with the Declaration of Helsinki. Written informed consent was obtained from all participants prior to inclusion.

### 2.3. SNP Selection and Genotyping

Candidate single nucleotide polymorphisms (SNPs) were selected from key genes involved in the m6A RNA modification pathway, including METTL3 (rs1139130), METTL14 (rs298982 (G > A)), FTO (rs9939609 (T > A)), ALKBH5 (rs1378602), and YTHDF1 (rs6011668). For each polymorphism, the genotype associated with poorer survival in the primary analyses and supported by published functional evidence was designated as the unfavorable (high-risk) genotype. Specifically, METTL3 rs1139130 (GG), METTL14 rs298982 (AA), FTO rs9939609 (AA), ALKBH5 rs1378602 (TT), and YTHDF1 rs6011668 (CC) were considered high-risk genotypes. These classifications were based on available evidence indicating that the corresponding alleles may alter gene expression or regulatory activity, thereby influencing m6A RNA methylation and downstream oncogenic pathways. Each unfavorable genotype was assigned one point in the construction of the cumulative genetic risk score. These variants were chosen based on three predefined criteria: (1) previous evidence of association with cancer susceptibility or prognosis; (2) predicted or experimentally validated functional relevance, including effects on gene expression, transcription factor binding, splicing, or regulatory activity; and (3) a minor allele frequency (MAF) greater than 5% in public population databases to ensure adequate statistical power. Genomic DNA was extracted from peripheral blood samples, and genotyping was performed using TaqMan allelic discrimination assays (Applied Biosystems, Foster City, CA, USA) according to the manufacturer’s instructions.

### 2.4. Genotyping Quality Control

To ensure the reliability of the genotyping data, several quality control procedures were implemented. DNA samples with inadequate quality or quantity were excluded before analysis. The overall genotyping call rate exceeded 98% for all investigated SNPs. Genotyping quality was assessed using standard quality-control procedures. The overall SNP call rate was 99.2%, with individual SNP call rates ranging from 98.7% to 99.8%. Approximately 10% of DNA samples (n = 23) were randomly selected for repeat genotyping as blinded duplicates, demonstrating 100% genotype concordance. Negative (no-template) controls were included in each PCR plate to monitor potential contamination, and no evidence of contamination was detected. Approximately 5–10% of randomly selected samples were genotyped in duplicate, and complete concordance was observed between duplicate assays. In addition, genotype distributions for each SNP were evaluated for conformity with Hardy–Weinberg equilibrium (HWE) using the chi-square goodness-of-fit test. No significant deviations from HWE were detected (all *p* > 0.05), supporting the reliability of the genotyping results.

### 2.5. Follow-Up and Outcome Measures

Follow-up was from the day of surgery until the end of life or the last follow-up assessment. Follow-up assessments included physical examination, imaging, and laboratory investigations, which were performed every 3 to 6 months for 2 years and then yearly afterward. The OS was measured as the interval between surgery and any cause of death, while DFS was measured as the interval between surgery and the first recurrence of the disease or death.

### 2.6. Genetic Risk Score Construction

An unweighted genetic risk score (GRS) was constructed to evaluate the cumulative effect of multiple m6A pathway polymorphisms on patient prognosis. For each SNP, the genotype associated with poorer survival in the primary analysis was defined as the unfavorable genotype. Individuals carrying an unfavorable genotype received one point, whereas those carrying the reference or low-risk genotype received zero points. The total GRS was calculated by summing the number of unfavorable genotypes across all selected SNPs, resulting in a cumulative score that reflected the individual’s overall genetic risk burden. Patients were subsequently categorized into tertiles according to their GRS. An unweighted scoring approach was selected because reliable external effect-size estimates for these polymorphisms in gastric cancer are currently unavailable. Furthermore, given the moderate sample size of the present study, equal weighting reduces the likelihood of overfitting and provides a simple, reproducible, and transparent method for evaluating cumulative genetic risk. Similar unweighted GRS models have been widely employed in candidate-gene association studies when validated weighting coefficients are lacking. Overall, the cumulative genetic risk score ranged from 0 to 5 and was initially categorized into tertiles according to its distribution in the study population: low-risk (GRS: 0–1), intermediate-risk (GRS: 2–3), and high-risk (GRS: 4–5). For Kaplan–Meier survival analyses and baseline comparisons, the score was additionally analyzed as a binary variable by combining the low- and intermediate-risk groups into a single low-risk category (GRS 0–3) and comparing them with the high-risk group (GRS 4–5). This approach was adopted to improve statistical power, simplify the visualization of survival curves, and facilitate clinical interpretation while preserving the primary tertile-based analysis for evaluating dose–response relationships.

Because only one representative SNP was analyzed for each candidate gene, linkage disequilibrium and haplotype analyses were not performed. The selected polymorphisms are located in distinct genomic regions, minimizing the likelihood of non-random allelic association among loci and supporting the assumption that each variant contributes independent genetic information to the cumulative genetic risk score.

### 2.7. Adjuvant Treatment

Postoperative adjuvant treatment was administered according to contemporary institutional protocols and international clinical practice guidelines, taking into account pathological stage, lymph node involvement, surgical margin status, patient performance status, and multidisciplinary team recommendations. Adjuvant therapy primarily consisted of fluoropyrimidine-based or platinum-containing chemotherapy, with selected patients also receiving chemoradiotherapy when clinically indicated. For statistical analyses, adjuvant treatment was categorized as **yes** or **no** based on whether patients received any postoperative systemic therapy with or without radiotherapy. This variable was included as a covariate in the multivariable Cox proportional hazards model to adjust for its potential impact on overall survival.

### 2.8. Statistical Analysis

Descriptive analysis was performed to provide summaries on the baseline characteristics of the included patients. The differences between the groups were tested using the chi-squared test for categorical variables, whereas the independent t-test or Mann–Whitney U test was adopted to test continuous variables. OS and DFS rates were evaluated through Kaplan–Meier estimation and compared using the log-rank test. The multivariate Cox proportional hazards regression model was conducted to investigate the association between the genotypes of the polymorphisms (genetic risk scores) and survival, adjusting for possible confounders such as age, gender, stage of the disease, lymph node metastasis, and adjuvant treatment. HRs and 95% CIs were calculated. The proportional hazards assumption was evaluated using Schoenfeld residuals and graphical inspection of log-minus-log survival plots. No significant violations of the proportional hazard assumption were observed. All statistical analyses were performed using SPSS software (Version 26.0; IBM Corp., Armonk, NY, USA).

## 3. Results

### 3.1. Patient Characteristics

A total of 226 patients with histologically proven gastric adenocarcinoma and undergoing curative gastrectomy surgery were recruited for this study. Their average age was 61 years (range: 34–82 years old), and among them, 142 (62.8%) were males. Based on the 8th edition of the AJCC staging system, their tumor stages were classified as stage I in 54 (23.9%), stage II in 78 (34.5%) and stage III-IV in 94 (41.6%) patients. Lymph node metastases existed in 137 patients (60.6%) and 158 of them (69.9%) received adjuvant treatment. There were no statistical differences in baseline data among risk groups (Table 1). For descriptive comparisons, patients belonging to the lowest and highest tertiles of the genetic risk score were compared, while those in the intermediate tertile were excluded to maximize the contrast between extreme genetic risk categories. This analysis therefore included 150 of the 226 study participants (75 low-risk and 75 high-risk patients).

### 3.2. Genotypes and Allele Distributions

The observed genotype distributions and minor allele frequencies are presented in Table 2. The MAFs ranged from 0.21 to 0.39, which is consistent with those reported in East Asian populations. None of the investigated polymorphisms deviated significantly from Hardy–Weinberg equilibrium (all *p* > 0.05), indicating satisfactory genotyping quality.

### 3.3. Genetic Risk Score Construction

A cumulative genetic risk score (GRS) was constructed using five functional SNPs in m6A RNA modification pathway genes (METTL3 rs1139130, METTL14 rs298982, FTO rs9939609, ALKBH5 rs1378602, and YTHDF1 rs6011668). For each polymorphism, the genotype associated with poorer survival was defined as the unfavorable genotype and assigned one point, whereas the remaining genotypes were assigned zero points. Consequently, the total GRS represented the sum of unfavorable genotypes across the five loci and ranged from 0 to 5, with higher scores indicating a greater cumulative genetic risk burden. Patients were subsequently categorized into tertiles according to the observed distribution of GRS values in the study population (low-, intermediate-, and high-risk groups) for survival analyses (Figure 1).

### 3.4. Association Between m6A Gene Variants and Survival

**Figure 2:**
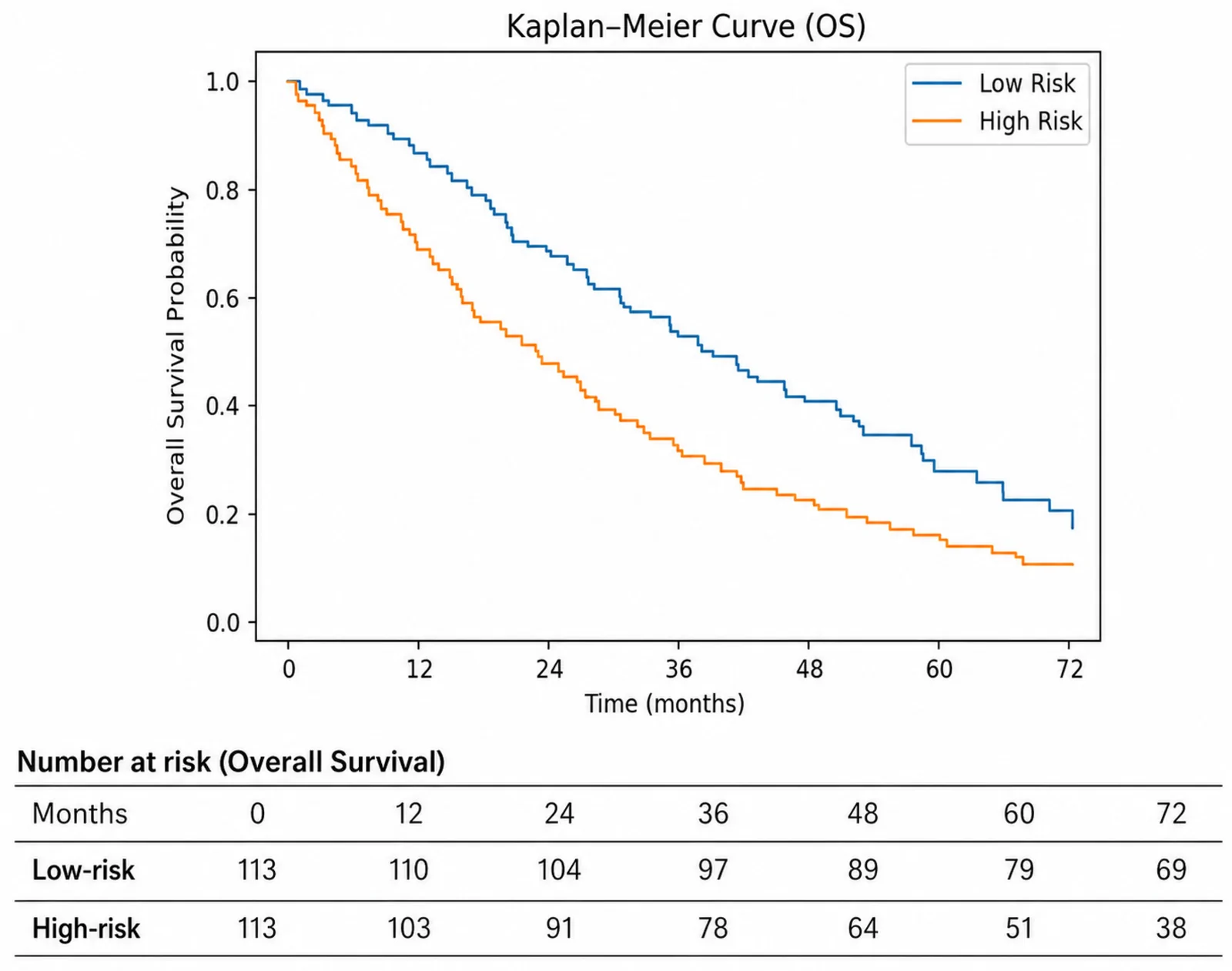
Kaplan–Meier curves for overall survival according to the genetic risk group. The numbers of patients at risk at each follow-up interval are displayed below the x-axis (patients were dichotomized into low-risk (GRS 0–3) and high-risk (GRS 4–5) groups for Kaplan–Meier analysis).

**Figure 3:**
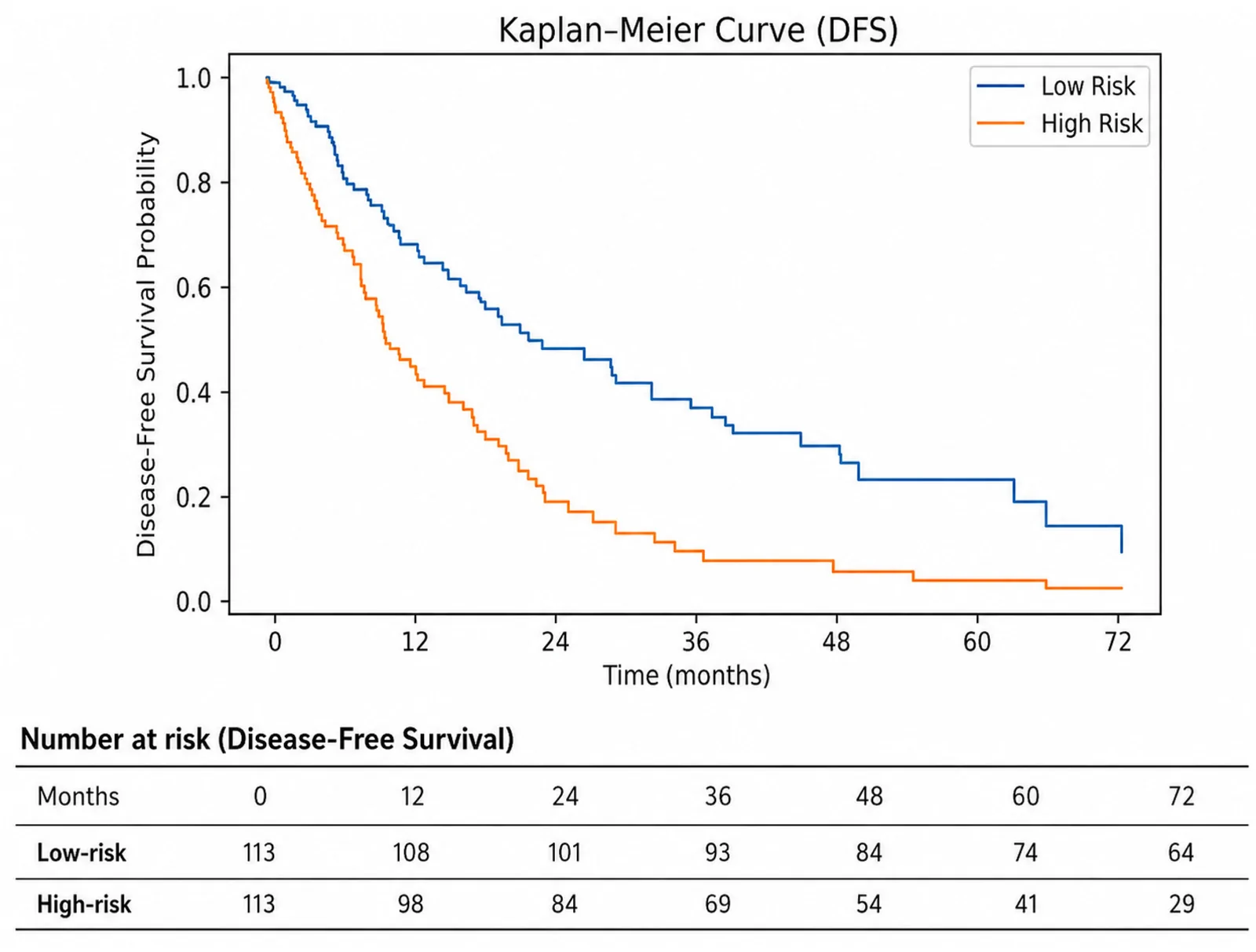
Kaplan–Meier curves for disease-free survival according to the genetic risk group. The numbers of patients at risk at each follow-up interval are displayed below the x-axis (Patients were dichotomized into low-risk (GRS 0–3) and high-risk (GRS 4–5) groups for Kaplan–Meier analysis).

The median follow-up duration was 38.0 months (interquartile range 24.0–56.0 months), with a maximum follow-up of 71.8 months. During follow-up, 78 patients (34.5%) died, and 92 patients (40.7%) experienced disease recurrence. The Kaplan–Meier survival analysis showed that patients with high-risk genotypes in m6A-related genes showed significantly lower OS and DFS compared with those with low-risk genotypes (*p* < 0.001 for both). Five-year OS rates were 68.2% and 41.5% in low- and high-risk groups, respectively (Figure 2). Likewise, the five-year DFS rates were 61.4% and 36.8% in low- and high-risk groups, respectively (Figure 3 and Table 3).

### 3.5. Multivariable Cox Regression Analysis

Before construction of the cumulative genetic risk score, each selected SNP was analyzed individually for its association with overall survival and disease-free survival. Individual variants demonstrated variable effect sizes, with some polymorphisms showing nominal associations with survival, whereas others exhibited weaker individual effects. Overall, the cumulative genetic risk score provided greater prognostic discrimination than any single polymorphism, suggesting that the combined contribution of multiple variants within the m6A pathway better reflects the genetic architecture underlying patient prognosis. Even after controlling for age, gender, cancer stage, lymph nodes, and adjuvant treatment, the high-risk gene polymorphisms were still significantly related to poor survival. Those in the high-risk category had a significantly higher hazard ratio for both mortality (HR: 1.87; 95% confidence interval: 1.28–2.74; *p* = 0.001) and recurrence (HR: 1.94, 95% CI: 1.36–2.78, *p* < 0.001) (Table 4).

### 3.6. Genetic Risk Score and Prognosis

The patients were categorized according to the tertiles of the cumulatively calculated genetic risk score. The worst outcome was observed in patients belonging to the third tertile, where there was significantly lower OS and DFS than in the second and first tertiles (*p* for trend < 0.001) (Table 5). Furthermore, a dose–response relationship was evident, implying that the more unfavorable the alleles, the worse the outcome. Stratification analysis showed that the role of m6A-associated genetic polymorphisms in the prognosis was higher in patients with advanced-stage (III–IV) cancer compared to the early stages. Among patients in the former category, there was significantly lower OS and DFS if they had high-risk genotypes (*p* < 0.001).

Subgroup analyses demonstrated that the prognostic value of the genetic risk score differed according to tumor stage (Table 6). Among patients with stage I–II disease, the genetic risk score showed a modest and non-significant association with overall survival (HR = 1.29, 95% CI: 0.82–2.03; *p* = 0.268) and disease-free survival (HR = 1.34, 95% CI: 0.88–2.05; *p* = 0.179). In contrast, among patients with stage III–IV disease, a high genetic risk score was independently associated with significantly poorer overall survival (HR = 2.41, 95% CI: 1.52–3.83; *p* < 0.001) and disease-free survival (HR = 2.58, 95% CI: 1.67–3.99; *p* < 0.001). Formal interaction analyses demonstrated a significant interaction between tumor stage and genetic risk score for both overall survival (*p* for interaction = 0.031) and disease-free survival (*p* for interaction = 0.018), indicating that the adverse prognostic effect of the genetic risk score was more pronounced in advanced-stage disease.

### 3.7. Association of Cumulative Genetic Risk Score with Survival

To account for multiple comparisons, *p* values from the individual SNP analyses were additionally adjusted using the Benjamini–Hochberg false discovery rate procedure. The cumulative genetic risk score remained significantly associated with both overall survival and disease-free survival after adjustment, whereas the results for individual SNPs should be interpreted as exploratory (Table 7).

### 3.8. Incremental Prognostic Value of the Genetic Risk Score

To determine whether the genetic risk score provided prognostic information beyond established clinicopathological factors, we compared two multivariable Cox regression models: a clinicopathological model including age, sex, tumor stage, lymph node status, and adjuvant therapy, and an extended model additionally incorporating the genetic risk score (Table 8). Addition of the genetic risk score improved model discrimination, with the C-index increasing from 0.741 to 0.772. Model fit was also improved, as indicated by a reduction in the Akaike Information Criterion from 812.4 to 804.1. Furthermore, the likelihood-ratio test demonstrated a statistically significant improvement in model performance after incorporation of the genetic risk score (Δχ^2^ = 10.1, *p* = 0.0015). These findings indicate that the genetic risk score provides prognostic information beyond established clinicopathological factors and improves the overall discriminatory performance of the multivariable survival model.

## 4. Discussion

This retrospective cohort study on 226 patients who received curative gastrectomy for gastric cancer showed that variations in genes involved in m6A RNA modification pathways have a significant correlation with patient survival. Patients with high-risk genotypes had worse OS and DFS regardless of existing clinical and pathological factors. In addition, there is a dose–response effect when looking at the cumulative risk score of the genetic risks involved.

In the last decade, the m6A epitranscriptomic mark has gained attention as an essential mechanism that regulates post-transcriptional gene expression through affecting RNA stability, translation efficiency, and cell differentiation [19]. Genetic and biochemical perturbations to the m6A machinery, including “writers” (METTL3, METTL14), “erasers” (FTO, ALKBH5), and “readers” (YTHDF1) proteins, have been found to be involved in carcinogenesis and tumorigenesis [20,21,22]. Here, we add to the existing literature by emphasizing that besides gene expression aberration, genetic polymorphisms in m6A-related genes can serve as biomarkers of poor prognosis.

Past investigations have primarily centered on the mRNA expression levels of m6A regulators in gastric cancer, as well as in other tumors, where the overexpression of METTL3 and YTHDF1, for example, has been shown to correlate with increased tumor proliferation and worse survival rates. Information about the relationship between genetic variants (SNPs) of these genes and the survival prognosis is, however, still sparse. This research project aims to fill this gap by investigating the effect of several genetic variants and developing an integrated prognostic risk score based on these findings.

Of particular interest is the finding that the predictive value of m6A-regulated mutations is more evident among patients with advanced tumors. These results imply that the aberration of RNA modifications might be more critical to tumor development and metastatic capability rather than the early onset of the disease. The study results also imply that individuals with advanced cancer could greatly benefit from molecular-based diagnostics and therapy strategies.

The advantages of the current research include a clearly defined cohort population, complete genetic profiling involving many crucial genes related to m6A modification, and reliable survival analysis with adjustments made for important covariates. However, there are a number of weaknesses that need to be mentioned. Firstly, the design of the study is retrospective, thus potentially causing a problem of bias. Secondly, the sample size used is rather small, which could affect the representativeness of the results obtained. Moreover, no functional assessment of identified SNPs was carried out, and the biological pathways involved in the process are yet to be identified.

Although previous studies have primarily focused on the expression profiles of m6A regulators, accumulating evidence suggests that inherited polymorphisms within these genes may also contribute to cancer susceptibility and clinical outcomes [23,24]. Functional variants in METTL3 have been associated with altered transcriptional activity and increased susceptibility to gastrointestinal malignancies, whereas polymorphisms in METTL14 have been linked to gastric cancer risk in Asian populations through their potential effects on RNA methyltransferase activity [25]. Likewise, common variants in FTO, particularly rs9939609, have been associated with cancer susceptibility and adverse prognosis in several malignancies, possibly through altered regulation of m6A demethylation and downstream oncogenic pathways [26]. Although evidence regarding ALKBH5 and YTHDF1 polymorphisms remains comparatively limited, emerging studies indicate that regulatory variants affecting these genes may influence tumor progression, immune regulation, and patient survival across several digestive system cancers, including gastric, colorectal, and hepatocellular carcinoma [27,28].

Our findings are generally consistent with these observations by demonstrating that inherited variation within the m6A regulatory pathway contributes to postoperative prognosis in gastric cancer. However, an important distinction of the present study is that we evaluated the combined prognostic effect of multiple polymorphisms rather than focusing on individual variants. Because each SNP exerted only a modest effect on survival, integrating them into a cumulative genetic risk score produced substantially stronger prognostic discrimination than would be expected from any single polymorphism alone. This pathway-based approach is biologically plausible because m6A modification is regulated through coordinated interactions among methyltransferases (“writers”), demethylases (“erasers”), and RNA-binding proteins (“readers”), whose collective dysregulation is more likely to influence tumor behavior than isolated genetic variants.

Despite these encouraging findings, published evidence regarding germline polymorphisms of m6A-related genes in gastric cancer remains relatively scarce. Most previous studies have investigated differential gene expression, somatic alterations, or epigenetic regulation rather than inherited variants [29,30]. Therefore, our study extends the current literature by suggesting that cumulative germline variation within the m6A pathway may provide clinically useful prognostic information following curative gastrectomy. Future multicenter studies with larger, ethnically diverse cohorts and functional experiments are warranted to validate these associations, identify causal mechanisms, and determine whether integrating m6A-related genetic risk scores with established clinicopathological factors can further improve personalized prognostic stratification.

Recent advances in artificial intelligence (AI) and machine learning (ML) have transformed biomarker discovery by enabling the integration of multi-omics, imaging, and clinical data to identify complex molecular signatures associated with cancer prognosis and therapeutic response. Such computational approaches are increasingly being applied in precision oncology to improve patient stratification, risk prediction, and treatment selection through the development of robust predictive models. In this context, inherited genetic variants affecting m6A regulators, including those identified in the present study, may represent valuable components of future multimodal prognostic frameworks. Integrating m6A-related polymorphisms with transcriptomic, epigenomic, and clinicopathological data using AI-driven analytical methods could substantially enhance prognostic accuracy and facilitate individualized therapeutic decision-making. These developments highlight the growing potential of combining molecular biomarkers with advanced computational methodologies to accelerate the clinical translation of precision oncology.

From a clinical perspective, the genetic risk score developed in this study has the potential to complement, rather than replace, established prognostic factors such as tumor stage, histological subtype, lymph node involvement, resection status, and molecular classification. Because germline genetic variants remain stable throughout life, they may provide additional prognostic information that is independent of tumor-related characteristics and could be incorporated into preoperative or postoperative risk assessment. Following rigorous external validation, the proposed score could contribute to identifying patients at higher risk of poor survival who may benefit from closer surveillance, individualized follow-up strategies, or more intensive adjuvant treatment. Furthermore, integrating this genetic score with conventional clinicopathological variables and emerging molecular biomarkers into comprehensive prognostic models may improve predictive accuracy and support precision oncology approaches in gastric cancer. Nevertheless, these potential applications remain speculative at present, and prospective multicenter studies involving independent and ethnically diverse populations are required to confirm the reproducibility, calibration, and clinical utility of the proposed risk score before routine implementation.

Although the selected polymorphisms were chosen based on previous evidence of potential functional relevance and biological plausibility, the present study was designed to evaluate their prognostic associations rather than their molecular consequences. Therefore, the proposed mechanisms linking these variants to gastric cancer progression remain inferential and are derived from published experimental studies on m6A regulators. The pathway schematic should be interpreted as a conceptual framework illustrating how altered m6A regulation may influence tumor biology rather than as direct evidence generated by the current investigation. Future studies integrating germline genotyping with gene-expression analyses, m6A methylation profiling, expression quantitative trait locus (eQTL) analyses, and laboratory functional assays will be necessary to determine whether these polymorphisms directly alter gene function or m6A regulatory activity and to elucidate the biological mechanisms underlying their prognostic associations.

An additional limitation of this study is that the proposed prognostic model and genetic risk score were developed and evaluated within a single study cohort and have not yet been externally validated in an independent patient population. Consequently, the model should be considered exploratory, and its predictive performance, calibration, and generalizability require confirmation in large, prospective, multicenter studies involving independent and ethnically diverse cohorts before routine clinical application can be recommended. A limitation of the present study is that formal linkage disequilibrium and haplotype analyses were not performed. However, because the genetic risk score incorporated one representative polymorphism from each of five distinct m6A-related genes located at different genomic loci, substantial linkage disequilibrium among the analyzed variants is unlikely. Consequently, the cumulative genetic risk score is expected to reflect the additive contribution of independent genetic loci rather than redundant information arising from correlated variants. Nevertheless, future studies evaluating multiple polymorphisms within individual genes should investigate linkage disequilibrium patterns and haplotype structures to determine whether combinations of intragenic variants provide additional prognostic information.

## 5. Conclusions

In summary, this study shows that alterations in m6A RNA modification pathway genes are independently related to worse survival outcomes in gastric cancer patients receiving curative gastrectomy surgery. Genotypes belonging to high-risk categories and higher cumulative genetic burden have been identified as independent predictors of poor overall and disease-free survival. The importance of m6A-related genetic assessment as a means for prognosis is supported by the results and may be included in individualized risk-stratification strategies. Further research is necessary for verification of these data and possible clinical applications of targeted therapy for m6A.

## Figures and Tables

**Table 1:** Baseline characteristics of patients in the lowest and highest tertiles of the genetic risk score (n = 150).

Variable	Total (n = 226)	Low-Risk (n = 75)	High-Risk (n = 75)	*p*-Value
Age (years), mean ± SD	61 ± 10.8	60.4 ± 11.2	61.7 ± 10.5	0.48
Male, n (%)	142 (62.8)	45 (60.0)	48 (64.0)	0.62
Stage (III–IV), n (%)	94 (41.6)	28 (37.3)	33 (44.0)	0.39
Lymph node positive, n (%)	137 (60.6)	42 (56.0)	47 (62.7)	0.37
Adjuvant therapy, n (%)	158 (69.9)	50 (66.7)	53 (70.7)	0.58

High-risk group = GRS 4–5; Low-risk group = GRS 0–3. Only patients in the lowest (n = 75) and highest (n = 75) tertiles of the genetic risk score were included in this comparison. Patients in the intermediate tertile (n = 76) were excluded to maximize contrast between extreme genetic risk categories.

**Table 2:** Genotype distribution, minor allele frequencies, and Hardy–Weinberg equilibrium.

Gene	SNP	Chromosome	Genomic Region	Genotype Distribution (n, %)	Minor Allele	MAF	HWE χ^2^	HWE P
METTL3	rs1139130	14	14q11.2	AA: 108 (47.8%), AG: 90 (39.8%), GG: 28 (12.4%)	G	0.323	0.47	0.492
METTL14	rs298982	4	4q26	GG: 119 (52.7%), GA: 82 (36.3%), AA: 25 (11.1%)	A	0.292	0.15	0.701
FTO	rs9939609	16	16q12.2	TT: 114 (50.4%), TA: 84 (37.2%), AA: 28 (12.4%)	A	0.310	0.26	0.609
ALKBH5	rs1378602	17	17p11.2	CC: 112 (49.6%), CT: 86 (38.1%), TT: 28 (12.4%)	T	0.314	0.09	0.761
YTHDF1	rs6011668	20	20q11.23	TT: 123 (54.4%), TC: 77 (34.1%), CC: 26 (11.5%)	C	0.286	0.63	0.427

HWE, Hardy–Weinberg equilibrium; MAF, minor allele frequency. HWE was evaluated using the chi-square goodness-of-fit test. No polymorphism showed a significant deviation from Hardy–Weinberg equilibrium (*p* > 0.05).

**Table 3:** Association of individual m6A-related gene polymorphisms with overall survival (OS) and disease-free survival (DFS).

Gene	SNP	Unfavorable Genotype	Frequency (%)	HR for OS (95% CI)	*p* Value	HR for DFS (95% CI)	*p* Value
METTL3	rs1139130	GG	31.4	1.32 (0.90–1.95)	0.152	1.39 (0.97–2.01)	0.074
METTL14	rs298982	AA	27.9	1.48 (1.01–2.18)	0.045	1.55 (1.08–2.24)	0.021
FTO	rs9939609	AA	24.8	1.56 (1.06–2.30)	0.024	1.63 (1.13–2.35)	0.011
ALKBH5	rs1378602	TT	29.2	1.37 (0.94–2.01)	0.103	1.42 (0.99–2.06)	0.062
YTHDF1	rs6011668	CC	26.5	1.61 (1.10–2.37)	0.017	1.69 (1.17–2.43)	0.006

**Table 4:** Multivariable Cox regression analysis for survival outcomes.

Variable	OS (HR, 95% CI)	*p*-Value	DFS (HR, 95% CI)	*p*-Value
High-risk genotype	1.87 (1.28–2.74)	0.001	1.94 (1.36–2.78)	<0.001
Age (>60 years)	1.32 (0.89–1.96)	0.16	1.28 (0.88–1.87)	0.19
Male sex	1.11 (0.75–1.65)	0.60	1.08 (0.74–1.59)	0.68
Stage (III–IV)	2.46 (1.63–3.71)	<0.001	2.59 (1.75–3.83)	<0.001
Lymph node positive	1.88 (1.22–2.88)	0.004	1.95 (1.30–2.93)	0.002
Adjuvant therapy	0.79 (0.53–1.18)	0.25	0.82 (0.56–1.21)	0.31

**Table 5:** Survival by genetic risk score tertiles.

Risk Group	5-Year OS (%)	5-Year DFS (%)	HR for OS (95% CI)	HR for DFS (95% CI)
Low tertile	70.1	63.2	Reference	Reference
Intermediate tertile	55.4	48.7	1.42 (0.91–2.21)	1.51 (1.00–2.28)
High tertile	39.8	34.5	2.03 (1.34–3.07)	2.11 (1.44–3.09)

**Table 6:** Subgroup analysis of the association between genetic risk score and survival according to tumor stage.

Tumor Stage	n	OS HR (95% CI)	*p* Value	DFS HR (95% CI)	*p* Value
Stage I–II	132	1.29 (0.82–2.03)	0.268	1.34 (0.88–2.05)	0.179
Stage III–IV	94	2.41 (1.52–3.83)	<0.001	2.58 (1.67–3.99)	<0.001
Interaction (*p* value)	—	0.031	—	0.018	—

**Table 7:** Individual associations of m6A-related gene polymorphisms with overall and disease-free survival, including false discovery rate (FDR)-adjusted *p* values.

	OS		DFS	
SNP	Raw P	FDR-Adjusted P	Raw P	FDR-Adjusted P
METTL3	0.152	0.190	0.074	0.123
METTL14	0.045	0.075	0.021	0.052
FTO	0.024	0.060	0.011	0.048
ALKBH5	0.103	0.155	0.062	0.103
YTHDF1	0.017	0.060	0.006	0.030

**Table 8:** Comparison of multivariable Cox regression models with and without the genetic risk score.

Model	Covariates Included	C-Index	Akaike Information Criterion (AIC)	Likelihood-Ratio χ^2^	Δχ^2^	*p* Value for Model Comparison
Model 1: Clinicopathological model	Age, sex, tumor stage, lymph node status, and adjuvant therapy	0.741	812.4	—	Reference	—
Model 2: Clinicopathological + genetic risk score	Age, sex, tumor stage, lymph node status, adjuvant therapy + GRS	0.772	804.1	10.1	10.1	0.0015

Abbreviations: AIC, Akaike Information Criterion; C-index, concordance index; GRS, genetic risk score. Note: The addition of the genetic risk score to the clinicopathological model improved discrimination, as demonstrated by an increase in the C-index from 0.741 to 0.772. Model fit was also improved, with a reduction in AIC from 812.4 to 804.1 and a statistically significant likelihood-ratio improvement (Δχ^2^ = 10.1, *p* = 0.0015).

## Data Availability

The original contributions presented in this study are included in the article. Further inquiries can be directed to the corresponding author.
